# Sea Anemone-Inspired Fluorosilicone Polyurethane Coating with Synergistic Low-Surface-Energy and Cationic Antibacterial Action for Static Antifouling

**DOI:** 10.3390/molecules31152717

**Published:** 2026-08-05

**Authors:** Shuiwang Jiang, Yuyi Zhu, Xiangfeng Chen, Hongyi Liu, Xuezhi Jiang, Yahao Zhang, Hui Gong, Ting Huang, Dengfeng Zeng, Quan Liu

**Affiliations:** 1 State Key Laboratory of Marine Corrosion and Protection, Luoyang Ship Material Research Institute, Xiamen 361100, China; jshuiw923@163.com (S.J.); wind8435201@163.com (X.C.); 512631878@qq.com (X.J.); zhangyahao725@163.com (Y.Z.); gonghui-1@163.com (H.G.); huangting1984@163.com (T.H.); 2 School of Architecture and Urban Planning, Chongqing University, Chongqing 400030, China; 3 Dongfang Electric (Fujian) Innovation Research Institute Co., Ltd., Fuzhou 350108, China; 4 School of Chemical and Biological Engineering, Institute of Zhejiang University-Quzhou, Zhejiang University, Hangzhou 310027, China

**Keywords:** marine antifouling, sea anemone-inspired, fluorosilicone polyurethane, cationic antibacterial, static antifouling

## Abstract

Conventional polydimethylsiloxane (PDMS)-based antifouling coatings, despite their inherent fouling-release capability, exhibit critically insufficient antifouling performance under static seawater conditions. Inspired by the synergistic physical–chemical defense strategy of sessile marine organisms, specifically sea anemones, which combine a physical mucus barrier with antimicrobial peptide secretion, the present work develops a multi-mechanism hybrid coating—designated as sea anemone-inspired fluorosilicone polyurethane—that integrates low-surface-energy physical antifouling and cationic antibacterial chemical antifouling. This coating system is constructed from silicone polyurethane (PDMS-PU), a cationic antibacterial moiety (PDMS-N^+^), and fluorinated functional monomers. Through systematic compositional optimization, an optimal formulation (P-4) is identified, which achieves a fracture elongation of 78.19%, a normal adhesion strength of approximately 2.5 MPa, a water contact angle of 120°, and a surface energy of 12.86 mN/m. Notably, its antibacterial rates against both Escherichia coli (*E. coli*) and Staphylococcus aureus (*S. aureus*) exceed 95%. The resultant coating uniquely synergizes low surface energy, potent antibacterial activity, excellent mechanical properties, and thermal stability, thereby enabling long-term and stable antifouling performance in static seawater environments. This work provides a crucial technological foundation for the engineering application and industrialization of green, durable marine antifouling coatings.

## 1. Introduction

Polydimethylsiloxane (PDMS) possesses intrinsically low surface energy [1], high chain flexibility [2], surface smoothness [3], compact structure [4], and excellent hydrophobicity [5], which enable effective removal of adhered fouling organisms under hydrodynamic shear in dynamic seawater environments, thereby exhibiting favorable fouling-release performance [6]. However, under static seawater conditions where sufficient hydrodynamic shear is absent, the antifouling capability of PDMS-based coatings deteriorates dramatically, failing to prevent the initial attachment and subsequent proliferation of marine microorganisms [7,8]. This pronounced deficiency in static antifouling performance has become a critical bottleneck limiting the practical application of PDMS coatings in static or low-speed marine equipment, such as buoys, offshore platforms, and ships at berth [8].

Fluorinated materials similarly exhibit low surface energy and offer superior mechanical strength and excellent corrosion resistance [9], rendering them potential candidates for antifouling coatings [10,11]. Nevertheless, the excessively high elastic modulus of most fluorinated polymers compromises the detachment efficiency of fouling organisms, as rigid surfaces tend to retain rather than release attached biofouls [12,13]. To reconcile the flexibility of silicones with the robust and water-resistant characteristics of fluoropolymers, fluorinated segments have been introduced into silicone matrices to construct fluorosilicone hybrid systems [12,14]. This hybrid strategy [15] enhances the mechanical integrity and hydrolytic stability of the resulting coatings while retaining the low-surface-energy physical antifouling advantage [16,17]. Nonetheless, relying solely on a single physical antifouling mechanism based on low surface energy—whether from silicones, fluorinated polymers, or their hybrids—remains inadequate to deliver satisfactory antifouling performance under truly static seawater conditions [18,19]. This is because a purely non-stick physical strategy, lacking external hydrodynamic assistance, cannot actively kill or repel microorganisms approaching the surface [17]. Therefore, an additional active antifouling functionality must be incorporated into fluorosilicone systems to compensate for the failure of physical antifouling under static conditions [20].

Remarkably, sessile marine organisms such as sea anemones (Actiniaria) maintain remarkably clean surfaces even after prolonged immersion in microbe-rich seawater [21]. The body surface of sea anemones secretes a mucus layer composed of glycoproteins and lipids, which forms a physical isolation barrier to prevent microbial colonization; meanwhile, their cnidocytes and epithelial cells can secrete antimicrobial peptides, lysozymes, and other active substances to kill attached microorganisms. The synergistic effect of physical barrier and chemical sterilization keeps the body surface clean for a long time in bacteria-rich seawater [22]. Inspired by this natural paradigm, integrating chemical antibacterial functionality with low-surface-energy physical antifouling offers a promising route to overcome the static antifouling bottleneck [2,23]. Cationic quaternary ammonium salts (QAS) represent a well-established class of broad-spectrum antibacterial agents [13] that disrupt microbial cell membranes via electrostatic interactions, thereby effectively inhibiting the adhesion and proliferation of marine microorganisms [24]. Grafting QAS moieties onto antifouling resin systems not only provides active chemical sterilization [11] but also modulates surface charge and hydrophilic–hydrophobic balance [24,25,26]. Hence, the integration of QAS with fluorosilicone low-surface-energy materials offers a viable biomimetic strategy for sea anemone-inspired static antifouling coatings.

Guided by this bioinspired design rationale, the present work synthesizes three functional monomers: a fluorinated monomer (PDMS-F, providing low surface energy for physical antifouling), a silicone polyurethane monomer (PDMS-PU, imparting flexibility and substrate adhesion), and a cationic antibacterial monomer (PDMS-N^+^, conferring broad-spectrum chemical antibacterial activity). By systematically tuning the copolymerization ratios of these three monomers, a multi-mechanism composite coating with robust mechanical properties and synergistic antifouling performance is constructed. The optimal formulation P-4 achieves a water contact angle of 120° and an ultra-low surface energy of 12.86 mN/m, outperforming typical fluorosilicone coatings (~115°, ~18 mN/m) and other fluorinated silicone systems (100.5–107.9°). Notably, the synergistic low-surface-energy and cationic antibacterial actions elevate antibacterial rates against *E. coli* and *S. aureus* to nearly 100%, substantially exceeding reported values for fluorinated zwitterionic silicone coatings (95.53% and 91.60%) [27] and rivaling quaternary ammonium-modified PDMS (98.9%) [23]. Moreover, unlike most existing studies that rely on biocidal additives or focus solely on dynamic fouling release, our bioinspired design tackles static antifouling failure via a dual-mechanism strategy rarely achieved without compromising mechanical properties or adhesion. This system is specifically engineered to overcome the static antifouling bottleneck of conventional PDMS coatings, establishing a key technological foundation for green, durable marine antifouling coatings.

## 2. Results and Discussion

### 2.1. Structural Characterization

**Figure 1:**
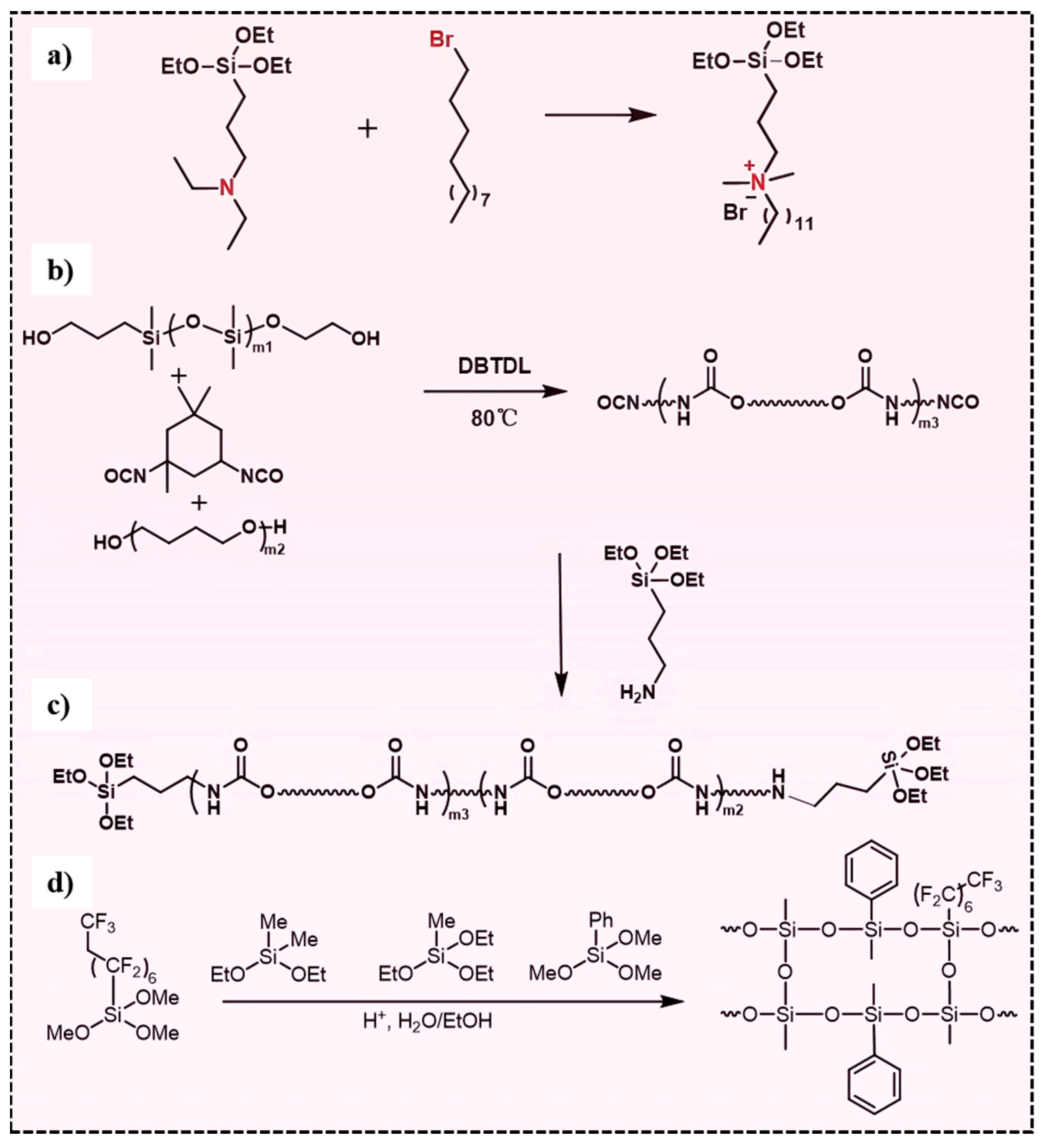
Preparation of sea anemone-inspired fluorosilicone polyurethane antifouling coating: (**a**) synthesis of PDMS-N^+^; (**b**) synthesis of PDMS-PU; (**c**) synthesis of PDMS-F; (**d**) synthesis of bioinspired antifouling resins.

The PDMS-based antifouling coating was synthesized through a polycondensation reaction, with the specific preparation process fully described in the Experimental Section (Figure 1). 

**Figure 2:**
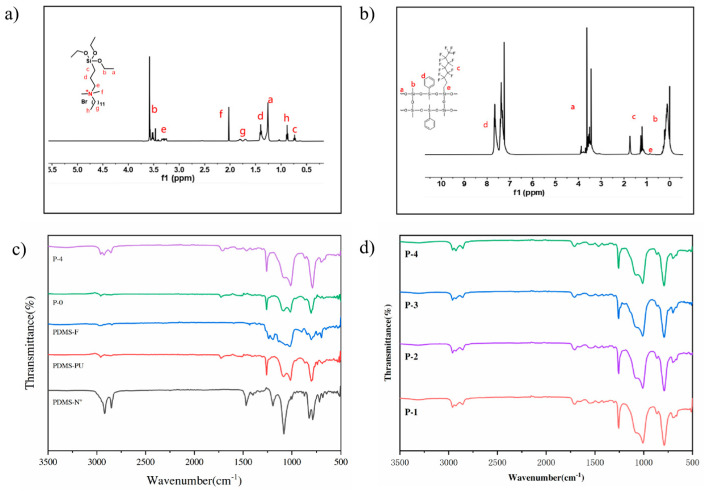
(**a**,**b**) Hydrogen nuclear magnetic resonance spectroscopy (^1^H NMR) of antifouling resin. (**c**,**d**) Carbon spectroscopy FTIR spectra of antifouling resin and its ingredients.

The molecular structures of the antifouling monomers PDMS-N^+^ and PDMS-F were thoroughly characterized using hydrogen nuclear magnetic resonance spectroscopy (^1^H NMR) and carbon spectroscopy (^13^C NMR) (Figure 2a,b). All characteristic peaks in the spectra were clearly identified: the quaternary ammonium salt N^+^-CH_3_ signal at δ 3.2–3.4 ppm for PDMS-N^+^ and the methylene signal associated with fluorinated alkyl groups at δ 1.5–1.6 ppm for PDMS-F confirmed their respective chemical structures. No significant residual peaks from the raw materials were observed, indicating high product purity and further verifying the successful synthesis of both functional monomers.

Functional group structure analysis of the antifouling monomer and the subsequently polymerized resin was performed using Fourier-transform infrared spectroscopy (FTIR) (Figure 2c). The vibration peaks at 800 cm^−1^ and 1008 cm^−1^ correspond to the bending and stretching vibrations of Si–O–Si bonds, respectively, indicating the presence of polysiloxane chain segments. The characteristic NCO peak at 2270 cm^−1^ observed in the IPDI (isophorone diisocyanate) feedstock was significantly attenuated in the PDMS-PU monomer spectrum and completely absent in the resin spectrum; instead, an N–H stretching vibration peak appeared in the 3300–3400 cm^−1^ range, demonstrating complete reaction between the isocyanate groups and hydroxyl groups to form a polyurethane structure, thereby confirming the successful synthesis of PDMS-PU monomers.

The infrared spectrum of the antifouling resin exhibits broad, intense absorption bands at 1050 cm^−1^ and 1130 cm^−1^, corresponding to the symmetric and asymmetric stretching vibrations of Si–O–Si bonds; absorption peaks at 1593 cm^−1^ and 1430 cm^−1^ are attributed to skeletal vibrations of Si–Ph (silicon–phenyl) groups, while the sharp absorption peak at 1270 cm^−1^ originates from bending vibrations of Si–CH_3_ (silicon–methoxy) groups (Figure 2d). The distinct absorption peak near 1220 cm^−1^ is identified as the characteristic stretching vibration of C–F bonds, indicating successful incorporation of fluorinated segments into the resin system. The co-occurrence of these characteristic peaks conclusively confirms the chemical structure of the designed antifouling resin and lays the foundation for subsequent surface property studies.

**Figure 3:**
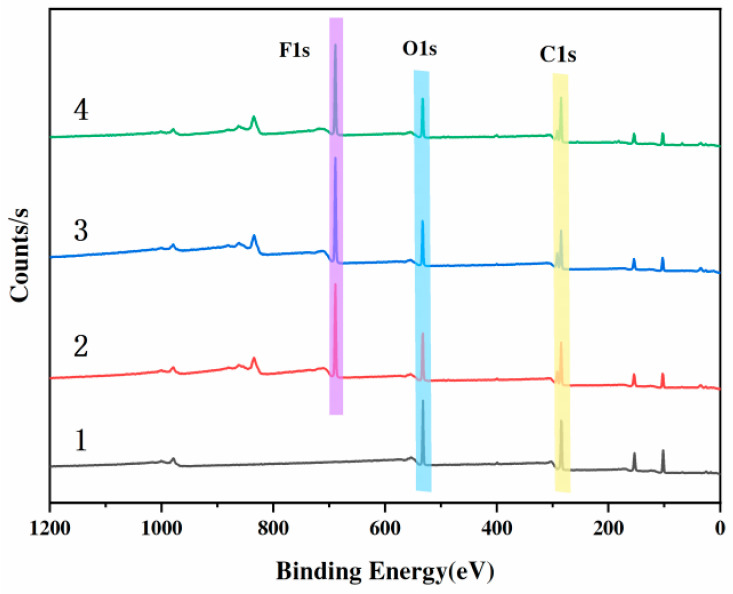
XPS data of sea anemone-inspired fluorosilicone polyurethane antifouling resin samples P-1, P-2, P-3, and P-4.

The elemental composition of the fluorinated silicone polyurethane coating surfaces was analyzed by X-ray photoelectron spectroscopy (Thermo, Waltham, MA, USA). As shown in Figure 3, no fluorinated resin component was incorporated into P-1. In the survey XPS spectra of P-2, P-3, and P-4, the binding energy peaks corresponding to C 1 s, N 1 s, O 1 s, and F 1 s appeared at approximately 283 eV, 399 eV, 531 eV, and 686 eV, respectively. These results confirm the successful preparation of the sea-anemone-inspired fluorosilicone polyurethane antifouling resin.

### 2.2. Surface Wettability

**Figure 4:**
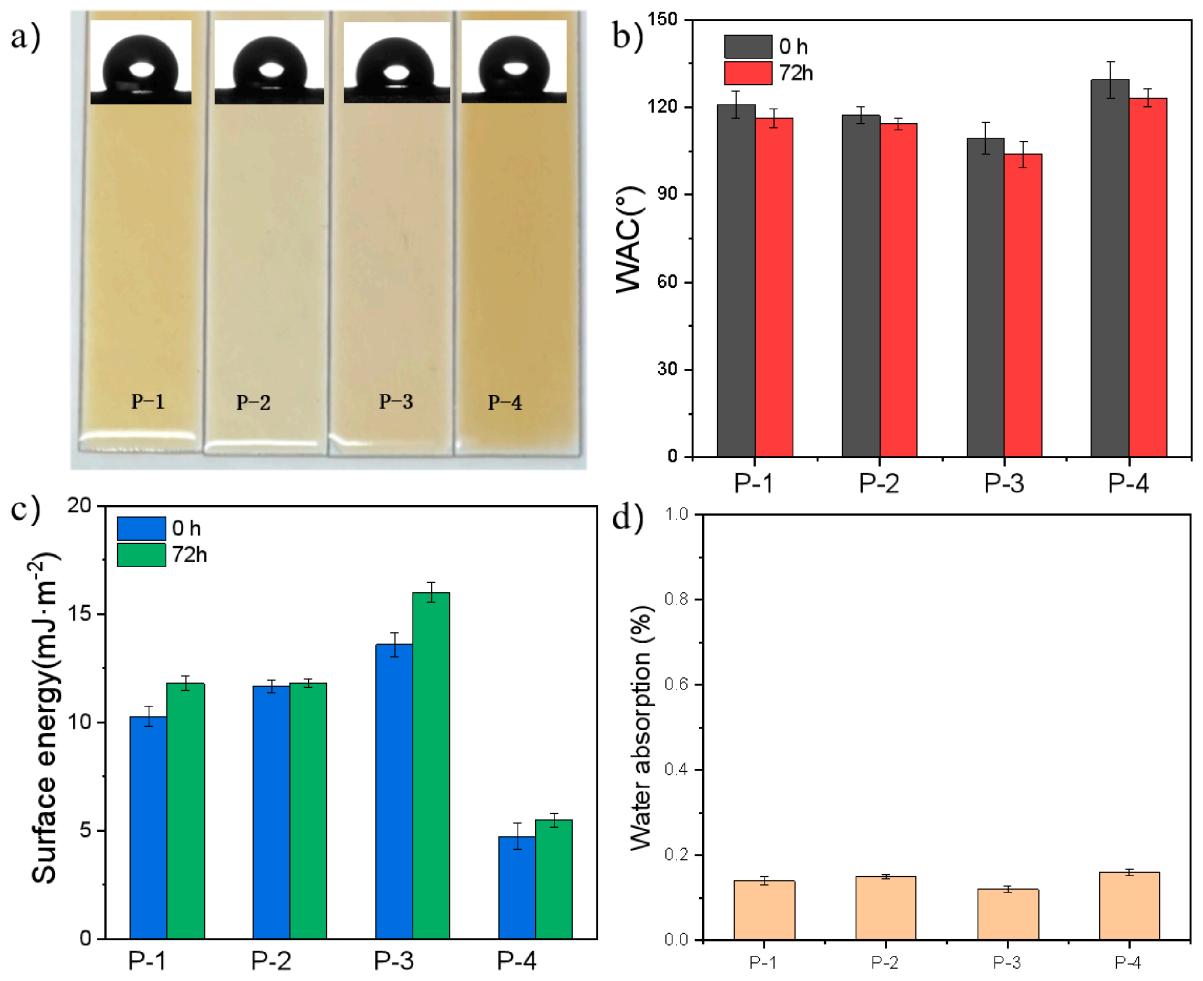
(**a**) The photos of the coating, (**b**) surface energy, and (**c**) water contact angle of P-1, P-2, P-3, and P-4 measured initially and after 72 h of immersion in water. (**d**) The water absorption rate of the coating after immersion in water for 72 h.

The surface wettability of the coatings was evaluated by static water contact angle (WCA) and diiodomethane contact angle measurements, from which the surface free energy (SFE) was calculated using the Owens-Wendt model [28]. Initial wetting performance evaluations indicated that all modified coatings manifested pronounced hydrophobic characteristics. The initial water contact angles for P-1, P-2, P-3, and P-4 measured 112°, 110°, 110°, and 120°, respectively (Figure 4a), corresponding to surface free energies of 14.233 mN/m, 16.077 mN/m, 16.629 mN/m, and 12.864 mN/m (Figure 4b). In comparison, the surface free energy of the unmodified pure polysilicone resin was 23.085 mN/m, confirming that functional monomer modification substantially reduces coating surface energy and enhances hydrophobicity (Figure 4b). Among the tested formulations, P-4 exhibited the most favorable initial hydrophobic performance, with its surface free energy reduced by 44.3% relative to pure PDMS. This behavior can be attributed to the surface migration and enrichment effect of fluorinated segments: owing to the high bond energy and low polarizability of C-F bonds, these segments spontaneously migrate toward the coating surface during the curing process, thereby forming a low-surface-energy hydrophobic layer. Furthermore, the incorporation of 30% PDMS-N^+^ in P-4 did not impede the migration of fluorine-containing chains; rather, it modulated phase separation within the polymer network, promoting uniform dispersion of the 5% fluorinated component on the surface and preventing the aggregation of fluorinated chains and the associated abnormal increase in surface energy observed in P-3 with 15% fluorine content.

**Figure 5:**
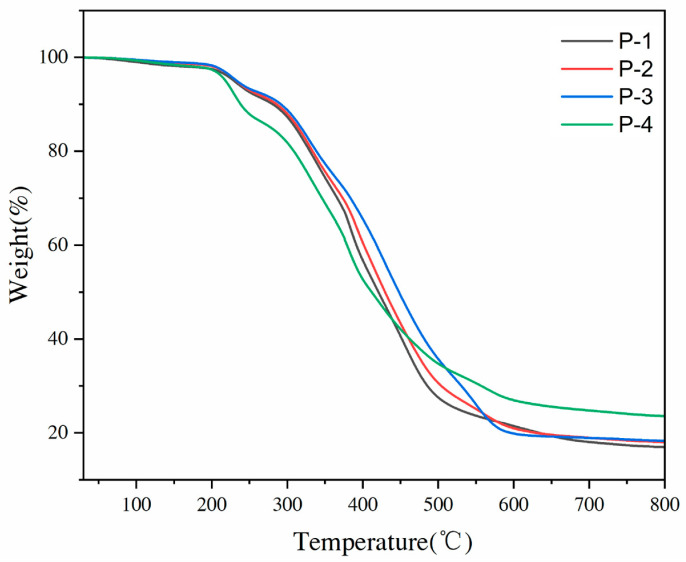
Thermogravimetric analysis (TGA) curves of a series of sea anemone-inspired fluorosilicone polyurethane antifouling coatings P-1, P-2, P-3, and P-4.

Wetting performance assessments conducted after 72 h of deionized water immersion revealed that the hydrophobic characteristics of the coatings were largely preserved. Notably, certain specimens exhibited surface restructuring, manifested as an increase in water contact angle accompanied by a decrease in surface free energy. The post-immersion water contact angles of P-1, P-2, P-3, and P-4 were 121°, 117°, 109°, and 129°, respectively, with corresponding surface free energies of 23.304, 10.272, 11.674, and 13.585 mN/m (Figure 5). Among them, P-4 demonstrated the most favorable hydrophobic stability, showing a pronounced increase in water contact angle (9°) and a marginal rise in surface free energy (0.721 mN/m). In aqueous environments, polymer surface chains undergo spontaneous rearrangement, leading to an increase in interfacial energy. Polar groups originally located in the interior (such as urethane bonds and quaternary ammonium cations) migrate toward the coating surface upon contact with water, while non-polar Si-O-Si segments and fluorocarbon chains migrate inward and become enriched, thereby reducing the water contact angle and increasing surface energy. Due to the high crosslinking density of the resin, this migration phenomenon is not pronounced, which helps the coating maintain a consistently low surface energy over long-term immersion in seawater.

In summary, the P-4 formulation combines the lowest initial surface free energy with superior hydrophobic stability under prolonged water immersion, with a water absorption rate of 0.16 wt% (Figure 4d). Its low surface energy significantly reduces the adhesion energy of marine fouling organisms, thereby promoting their release under hydrodynamic shear forces. Concurrently, the stable hydrophobic interface ensures sustained antifouling performance, providing a fundamental basis for the coating’s long-term serviceability in static marine environments.

### 2.3. Thermal Stability

Thermal stability is a critical performance criterion for marine antifouling resins, as coatings in service are frequently subjected to thermal exposure from solar radiation in tropical regions, engine heat, or elevated seawater temperatures. Insufficient thermal resistance can induce premature degradation of the polymer matrix, thereby compromising surface wettability, mechanical integrity, and long-term antifouling efficacy. The thermal behavior of the bioinspired fluorosilicone polyurethane antifouling resins (P-1 to P-4) was evaluated by thermogravimetric analysis (TGA). As shown in Figure 5, the thermal decomposition of all four samples occurred predominantly within the temperature range of 250–600 °C, characterized by a significant mass loss that corresponds to the major thermal degradation events of the polymer networks. Although high-temperature resistant coatings typically require structural and performance stability at substantially higher temperatures, the present series of resins does not meet the criteria for such extreme-service applications. Nevertheless, from the perspective of conventional functional materials, the thermal profiles of P-1 through P-4 are consistent with standard thermomechanical testing requirements, rendering them suitable for further performance evaluation and application development under typical marine service conditions.

### 2.4. Mechanical Properties

**Figure 6:**
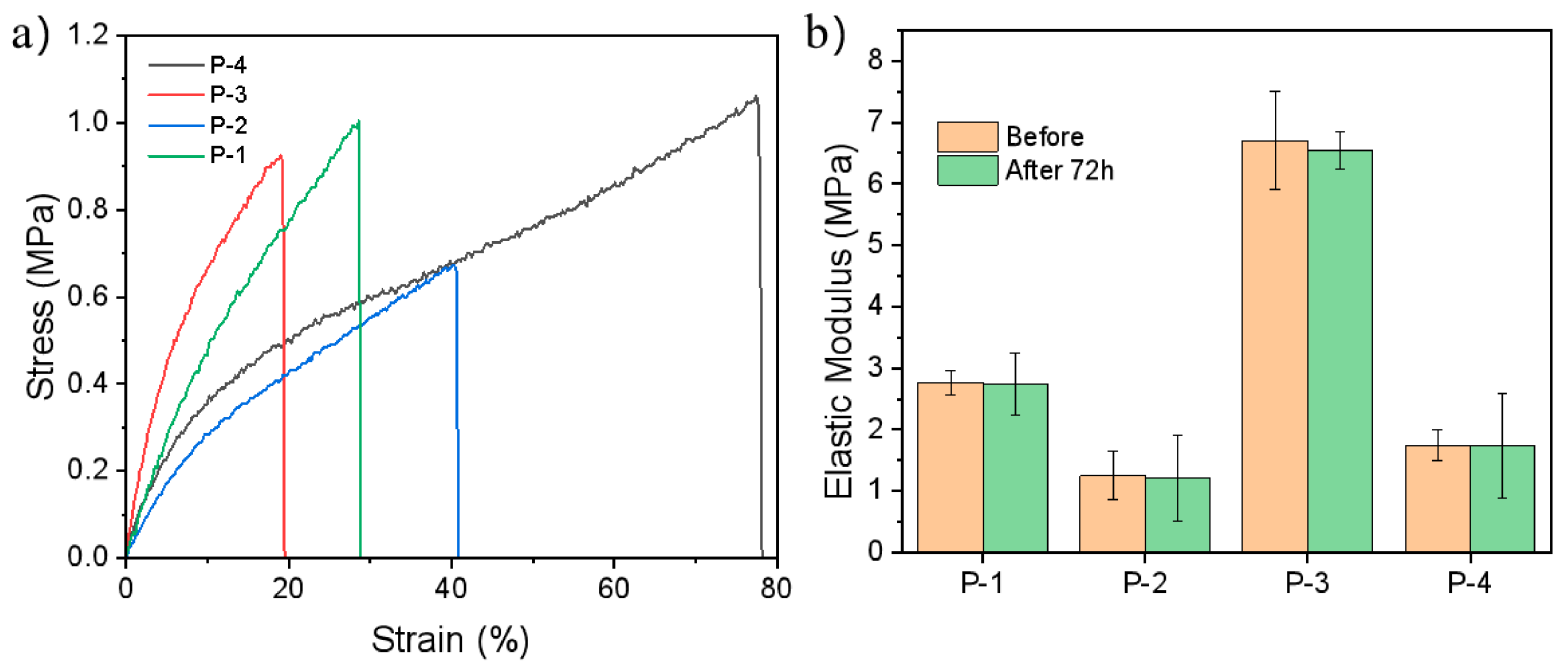
(**a**) Tensile properties (tensile strength and elongation at break) of the series of sea anemone-inspired fluorosilicone polyurethane antifouling resins. (**b**) The modulus of the coating before and after a 72-h immersion in water.

Polymers P1, P2, and P3 incorporate only 15% cationic monomers, leading to an insufficient number of crosslinking sites that detrimentally affect the overall material performance. In contrast, P-4 demonstrates an elevated cationic monomer content of 30%. Its quaternary ammonium siloxane side chains possess numerous alkoxysilane groups, which engage in siloxane condensation reactions with silicon hydroxyl groups generated during TEOS hydrolysis throughout the curing process, thereby moderately augmenting the system’s crosslink density. Compared to P-1 and P-2, which exhibit inadequate mechanical strength owing to deficient crosslinking points, and P-3, which displays excessive crosslinking and embrittlement due to high fluoropolymer content, P-4 attains an optimized crosslink density. This equilibrium avoids both the low strength associated with insufficient crosslinking and the restricted molecular chain mobility induced by over-crosslinking, consequently yielding a modest enhancement in macroscopic tensile strength. In P-4, PDMS-PU comprises 60% of the composition; the alternating architecture formed by polyurethane hard segments (derived from IPDI units) and silicone soft segments confers exceptional flexibility upon the siloxane backbone. Furthermore, urethane bonds within the polyurethane establish both intramolecular and intermolecular hydrogen bonds capable of reversible rupture and reformation under external stress, effectively dissipating mechanical energy and improving toughness. Conversely, the heightened fluoropolymer content (15%) in P-3 introduces a substantial number of rigid C-F bonds that constrain silicone chain mobility, impeding chain extension and precipitating a marked reduction in elongation at break. P-1 lacks fluorinated constituents and does not integrate rigid unit reinforcements; P-2, despite undergoing minor fluorination modifications, is hampered by insufficient cationic monomers—neither material achieves a simultaneous combination of high strength and favorable elongation (Figure 6a,b). By contrast, P-4 accomplishes moderate reinforcement through the maintenance of a 5% fluorine content coupled with a highly flexible polyurethane matrix, culminating in superior fracture elongation.

Due to the addition of F-containing functional units, the P-3 sample exhibited significantly superior hardness and modulus compared to other groups, reaching 3H and 6.7 MPa, respectively (Table 1). This is likely attributed to the strong polarity of C-F bonds, which promotes the extension and orderly arrangement of originally coiled polysiloxane chains, forming a structurally regular framework. Under tensile stress, this structure undergoes “strain-induced crystallization,” enhancing both the resin’s hardness and modulus. Consequently, this sample demonstrates better wear resistance in daily applications. However, the high hardness and modulus also compromise material toughness; under alternating seawater impacts, stress cannot be effectively dissipated through chain segment movement, increasing susceptibility to microcracks and coating delamination. In contrast, the P4 sample exhibits a modulus of 1.743 MPa and hardness of 2H, demonstrating balanced mechanical properties. Its molecular chain design enables stress buffering through chain deformation, sliding, and entanglement under external forces, combining robust strength with excellent deformability (Table 1). This mechanical equilibrium allows the material to better withstand prolonged water erosion and cyclic loads typical of marine coatings, ensuring adhesion and durability while effectively mitigating early failure caused by stress concentration, making it particularly suitable for applications in harsh marine environments.

### 2.5. Adhesion Performance

**Figure 7:**
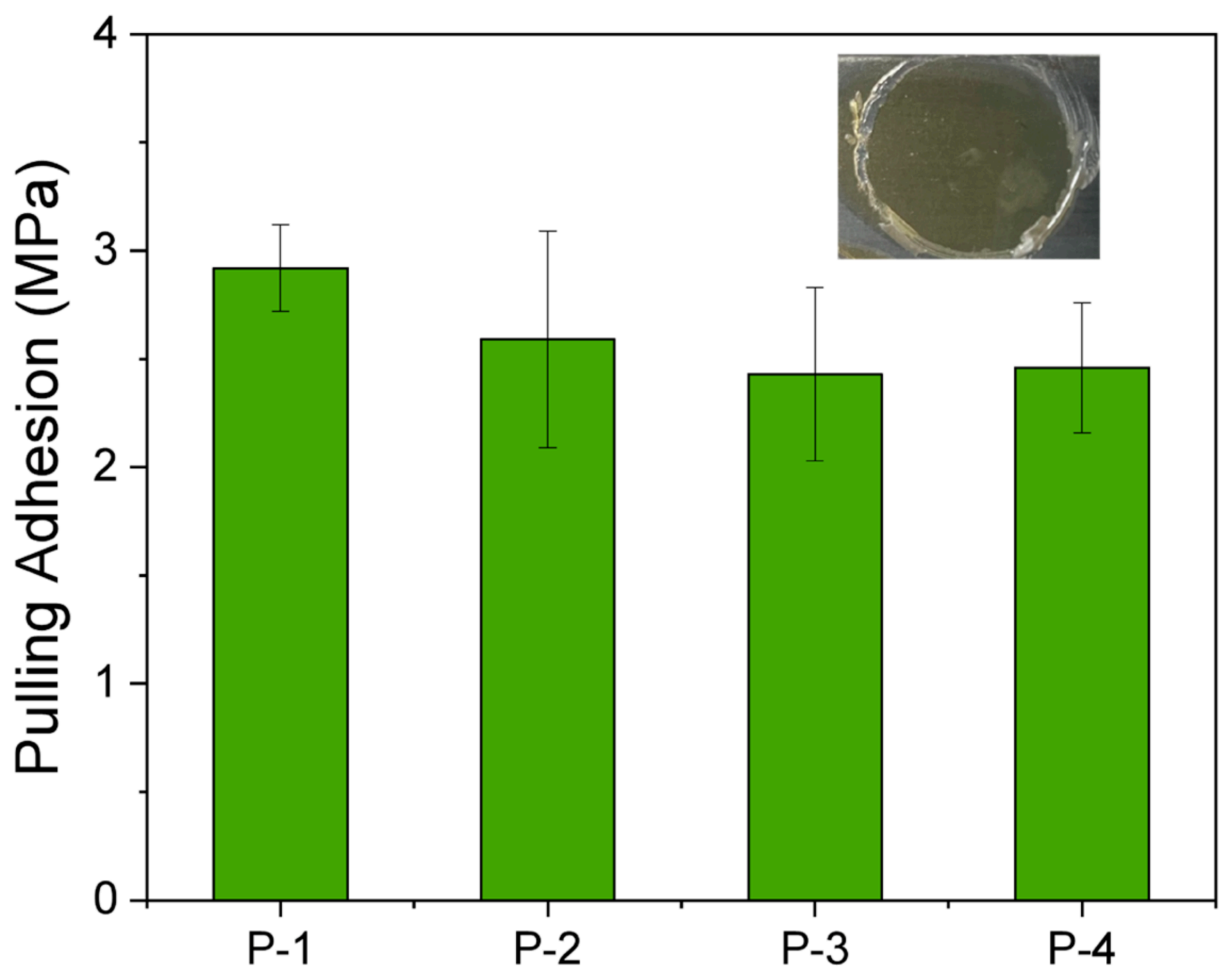
Adhesion of the antifouling coating. The inset shows the post-test photo, with traces of the coating remaining on the bottom layer.

Strong adhesion between the coating and the substrate is a prerequisite for the long-term service of marine antifouling coatings [25]. Insufficient adhesion leads to coating detachment under repeated hydrodynamic scouring and biofouling attack, ultimately resulting in complete loss of antifouling protection [26]. Therefore, the normal adhesion strength of the coatings was systematically evaluated in this work (Figure 7).

Pull-off test results demonstrate that the normal adhesion strength of all four modified PDMS composite coatings exceeded 2.5 MPa, significantly surpassing that of pure PDMS substrates (usually <1 MPa), thereby confirming that PDMS-PU and PDMS-N^+^ modifications effectively enhance the interfacial bonding between silicone coatings and tinplate substrates (Figure 7). Specifically, in systems P1-P3, the PDMS-N^+^ content was fixed at 15%, with performance optimized by adjusting the ratio of PDMS-PU to PDMS-F: the urethane polar groups in the polyurethane segments form strong hydrogen bonds with metal surface hydroxyl groups, while uniform dispersion of trace fluorine components improves resin curing density and fills microscopic pores at interfaces, resulting in reliable adhesion strength across all samples. In system P4, increasing PDMS-N^+^ content from 15% to 30% further elevated overall coating polarity due to the abundant polar sites of quaternary ammonium cations, leading to enhanced adhesion (Figure 7). The PDMS-PU content progressively decreased from 80% to 60% across samples P1–P4. Compared to sample P1, reduced urethane polar group proportions slightly diminished adhesion performance, highlighting the critical role of PDMS-PU components. Comprehensive analysis reveals significant variations in mechanical properties among samples, which are likely attributable to differences in chemical composition and molecular structure. For instance, the types and quantities of polar groups in the chemical composition directly influence interfacial interactions, while the arrangement of molecular structures determines the coating’s density. These findings provide crucial experimental insights for optimizing coating formulations, enabling enhanced performance through precise regulation of component ratios and introduction of novel functional groups—all while maintaining high adhesion.

### 2.6. Antibacterial Performance

**Figure 8:**
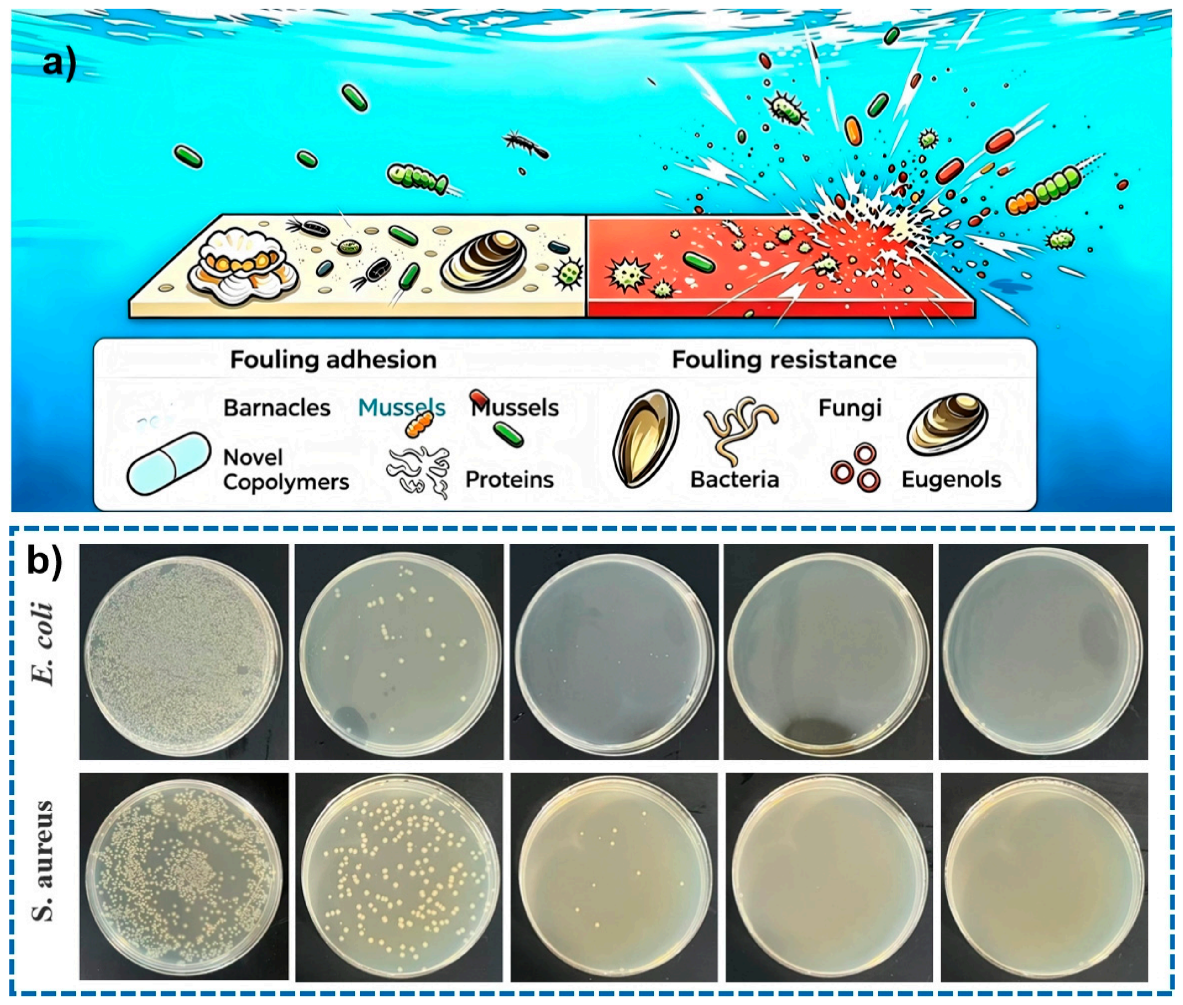
(**a**) Schematic illustration of the antifouling mechanism of a series of sea anemone-inspired fluorosilicone polyurethane antifouling coatings; (**b**) comparison of antibacterial performance between the developed coatings and the PDMS control coating (the figures show the coated plates of samples PDMS, P1, P2, P3, and P4, respectively).

The initial phase of marine biofouling predominantly involves microbial adhesion and biofilm formation, with Gram-negative *E. coli* and *S. aureus* representing characteristic strains implicated in biofilm development (Figure 8a). Consequently, these two bacterial species were selected to assess the anti-adhesion and antibacterial properties of the coatings with a co-culture time of 24 h. On the pure PDMS control coating, the adherent bacteria are 44.03 CFU/cm^2^ for *E. coli* and 7.86 CFU/cm^2^ for *S. aureus*. For *E. coli*, the CFU/cm^2^ at P1, P2, P3, and P4 are, respectively, 0.53, 0.14, 0.01, and 0 CFU/cm^2^. For *S. aureus*, the CFU/cm^2^ at P1, P2, P3, and P4 are, respectively, 1.65, 0.17, 0, and 0 CFU/cm^2^. Pure PDMS and the P-1 sample exhibited limited antibacterial efficacy and elevated bacterial adhesion rates. However, with an increase in the proportion of fluorinated segments in PDMS-F and the concentration of quaternary ammonium cations in PDMS-N^+^, bacterial attachment on the surfaces of P-2, P-3, and P-4 coatings markedly decreased, with P-4 demonstrating the most potent antibacterial activity (Figure 8b).

An in-depth mechanistic analysis indicates that quaternary ammonium cations function as the primary antibacterial sites. The underlying principle involves PDMS-N^+^ grafted onto the polymer backbone, which substantially enhances the surface charge density of the coating. These positive charges effectively capture negatively charged bacterial cell membranes via electrostatic adsorption, compromising the structural integrity of the phospholipid bilayer and inducing leakage of essential intracellular components, thereby leading to bacterial inactivation [29]. Furthermore, the incorporation of fluorinated constituents into the coating leverages their low-surface-energy characteristics to attenuate interfacial adhesion between bacteria and the coating surface, thereby inhibiting initial bacterial colonization. This design achieves a synergistic combination of physical anti-adhesion and chemical sterilization, collectively enhancing the antibacterial performance of the coating. P-1 contains only 15% PDMS-N^+^ and lacks fluorinated resin components; due to the absence of an anti-adhesion mechanism and a limited surface cation concentration, its antibacterial efficacy is substantially impaired. P-2 and P-3 incorporate 5% and 15% fluorinated monomers, respectively, which effectively reduce surface adhesion and suppress initial bacterial attachment. However, P-3 maintains a relatively high cation concentration (15%), which restricts the availability of active antibacterial groups and consequently limits further improvement in bactericidal efficiency. In contrast, P-4 increases the PDMS-N^+^ content to 30%, significantly elevating the surface charge density and amplifying the number of available sites for antibacterial interaction. Simultaneously, the incorporated 5% trace fluorinated resin retains low surface energy and anti-adhesion properties. The synergistic action of the two mechanisms yields antibacterial rates approaching 100% against both tested strains, significantly outperforming the majority of previously reported systems (which typically fall within 95–98%), thus underscoring the novelty and superiority of our bioinspired design. (Figure 8b).

The aforementioned results illustrate that the chemical bactericidal effect of high-concentration quaternary ammonium cations operates synergistically with the physical anti-adhesion properties conferred by fluorinated segments. P-4 effectively suppresses surface biofilm formation, thereby preventing subsequent adhesion of macrofouling organisms at the source and endowing the coating with superior static antifouling performance.

## 3. Experimental Section

### 3.1. Materials

Hydroxypropyl-terminated polydimethylsiloxane (HPT-PDMS, Mw = 2000 g/mol) was supplied by Jiande High-Tech Polymer New Materials Co., Ltd. (Hangzhou, China) and was dried under vacuum at 60 °C for 2 h prior to use. Isophorone diisocyanate (IPDI, 99%), 1H,1H,2H,2H-perfluorooctyltrimethoxysilane, methyltrimethoxysilane, dimethyldimethoxysilane, and eugenol (En, 99%) were purchased from Aldrich (Shanghai, China) and used as received. Dibutyltin dilaurate (DBTDL, 95%), N,N-diethyl-3-aminopropyltrimethoxysilane, dodecyl bromide, and tetrahydrofuran (THF, 99%) were obtained from Aladdin (Shanghai, China). THF was dried over molecular sieves before use, while all other reagents from Aladdin (Shanghai, China) were used without further purification. 2,2′-Azobisisobutyronitrile (AIBN) was purchased from Macklin (Shanghai, China) and recrystallized prior to use. A commercial PDMS elastomer (Sylgard 184, used as a control) was purchased from Dow Corning (Michigan, MI, USA) and used as received. All other reagents were used without further purification unless otherwise stated.

### 3.2. Material Synthesis and Preparation

#### 3.2.1. Synthesis of PDMS-N^+^

A mixture of N,N-diethyl-3-aminopropyltrimethoxysilane (APTES, 11.77 g, 50 mmol) and dodecyl bromide (12.462 g, 50 mmol) was placed in a single-neck round-bottom flask with acetonitrile (40 mL) as the solvent. The reaction was stirred at 85 °C for 24 h. The resulting solution was partially concentrated using a rotary evaporator at 50 °C for 8 min to remove most of the solvent. The crude product was washed three times with alternating diethyl ether and ethyl acetate to remove unreacted monomers (Figure 1a).

#### 3.2.2. Synthesis of PDMS-PU

The PDMS-PU prepolymer was synthesized via polycondensation (Figure 1b). An excess of IPDI was added dropwise to a solution of HT-PDMS and PTMG in the presence of an organotin catalyst to form a polyurethane prepolymer, followed by end-capping with excess KH-550. Specifically, a mixture of HT-PDMS (7.50 g, 3.75 mmol) and PTMG (3.75 g, 3.75 mmol) was placed in a three-neck round-bottom flask purged with nitrogen, using xylene (40.00 g) as the solvent. The mixture was stirred at 80 °C under nitrogen to form a homogeneous solution. Subsequently, IPDI (2.17 g, 9.75 mmol) and DBTDL (0.05 g) were added dropwise as the polyurethane hard segment and catalyst, respectively (NCO/OH molar ratio = 1.30:1). The reaction was continued for 2 h to obtain a transparent prepolymer solution. Then, excess KH-550 (1.10 g, 5 mmol) was added to react with the remaining ~NCO groups (~4.50 mmol) at 80 °C for another 2 h.

#### 3.2.3. Formulation and Curing of Bioinspired Antifouling Resins

Several formulations of bioinspired antifouling resins were designed in this work. According to the compositions listed in Table 2, four bioinspired antifouling resins were synthesized and designated as P-1, P-2, P-3, and P-4, respectively. Dibutyltin diacetate (0.08 wt%, listed in Table 2) was used as the curing catalyst for siloxane condensation, which was not clearly described in the original text. The coatings were cured at 25 °C with a relative humidity of 50% for 24 h. After room-temperature curing, the samples were placed in a vacuum oven at 60 °C for 2 h to completely remove residual solvents, with a thickness of 200 μm.

### 3.3. Evaluation of Physicochemical Properties of the Coatings

#### 3.3.1. Measurement of Tensile Properties 

Tensile tests of the polymer coating samples were performed using a Zwick/Roell Z020 universal testing machine. The polymer samples were cut from a polytetrafluoroethylene (PTFE) mold (30 mm × 30 mm × 3 mm) into small dumbbell-shaped specimens with dimensions of 25 mm × 2 mm × 0.25 mm. For each specimen, five individual tensile tests were recorded, and the average values were reported. The Young’s modulus of the specimens was calculated using the accompanying software.

#### 3.3.2. Normal Adhesion Strength Measurement

The coating samples were first applied onto tinplate substrates (25 mm × 25 mm × 250 μm). Subsequently, an epoxy adhesive was coated onto the surface of the dried coating. A cylindrical aluminum stub (dolly) was then adhered to the coated surface using the epoxy adhesive. After standing at room temperature for 24 h, the aluminum stub was firmly bonded to the tinplate, with a circular contact area of 16 mm in diameter. The normal adhesion strength was measured using a BGD500 digital semi-automatic pull-off tester (Biuged, Guangzhou, China) at a constant pulling speed of 1 MPa·min^−1^ at 25 °C. Five measurements were performed for each sample, and the average value was calculated.

#### 3.3.3. Hardness Measurement

The coating samples were first applied onto tinplate substrates (25 mm × 25 mm × 250 μm), and the panels were placed horizontally. Pencil hardness tests were performed. A pencil was pressed onto the coating surface at a 45° angle under a load of 500 g. The pencil hardness was determined by progressively increasing the hardness of the pencils drawn across the coating surface until the first defect appeared.

#### 3.3.4. Surface Wettability Measurement

Contact Angle (CA) and Surface Free Energy (SE): The contact angle of the coatings was measured using a CAM 200 optical contact angle goniometer (KSV Instruments Co., Ltd., Helsinki, Finland) with a droplet volume of 2 µL at room temperature. Contact angles were measured at least at five different positions on each coating, and the average value was calculated. The surface free energy of the coatings was determined using the Owens–Wendt–Rabel–Kaelble (OWRK) method by measuring the contact angles of deionized water and diiodomethane on the coating surfaces. Likewise, after immersing the coatings in water for 72 h, the contact angle and surface free energy were measured using the same procedure.

#### 3.3.5. Thermal Stability Measurement

The thermal stability of the coatings was evaluated by thermogravimetric analysis (TGA 309) (NETZSCH, Selb, Germany). Samples were heated from 30 °C to 800 °C at 10 °C/min under a nitrogen atmosphere, and the weight loss was recorded.

#### 3.3.6. Antibacterial Performance Measurement

The antibacterial performance of the coating samples against Escherichia coli and Staphylococcus aureus was evaluated using the inhibition zone method combined with microscopic observation. Mueller–Hinton (MH) agar plates were prepared. A sterile cotton swab was dipped into the bacterial suspension and evenly streaked across the MH agar surface three times, rotating the plate by 60° between each streaking, followed by a final smear along the inner edge of the plate. Antimicrobial susceptibility test discs were immersed in the prepared resin sample solutions and air-dried at room temperature for 3–5 min. The dried discs were then placed onto the agar surface using sterile forceps, ensuring firm contact between the disc and the agar. The plates were incubated at 37 °C for 16–24 h. After incubation, the formation of inhibition zones was observed, and the diameters of the inhibition zones were measured using a vernier caliper.

The antibacterial performance of the coating samples against Escherichia coli and Staphylococcus aureus was evaluated using the LB plate counting method combined with microscopic observation. First, glass slides (18 mm × 18 mm) coated with the sample coatings (18 mm × 18 mm) were placed into 20 mL of bacterial suspension (10^8^ CFU/mL) and incubated under shaking at 37 °C for 24 h. Subsequently, the coated glass slides were removed, and the uncoated side of each slide was gently rinsed with sterile PBS buffer. The rinsed slides were then transferred into 20 mL of sterile PBS buffer and subjected to ultrasonic oscillation for 15 min to detach the adherent *E. coli* and *S. aureus* from the coating surface. The resulting sonicated solution was diluted 1000-fold, and 20 μL of the diluted solution was inoculated onto the surface of LB agar plates, followed by incubation at 37 °C for 24 h. Finally, the number of bacterial colonies on the LB agar plates was counted using the LB plate counting method. The number of adherent colonies on the control PDMS coating was recorded as *N*_1_, while that on the experimental coating samples was recorded as *N*_2_. The antibacterial adhesion efficiency (*AE*) was calculated according to the following equation:

$$AE=(1-\frac{N1}{N2})\times 100\%$$

#### 3.3.7. Synthesis of PDMS-F

The fluorinated resin was prepared via hydrolysis reaction (Figure 1c). 1H,1H,2H,2H-Perfluorooctyltrimethoxysilane, methyltrimethoxysilane, and dimethyldimethoxysilane were mixed in a single-neck flask at a molar ratio of R/Si = 1.4 and F/Si = 0.4. The mixture was dissolved in a solution of water (pH = 3) and xylene, and the hydrolysis reaction was carried out at 60 °C for 2 h. The resulting solution was concentrated using a rotary evaporator at 60 °C for 20 min to remove the solvent.

## 4. Conclusions

In summary, drawing inspiration from the synergistic physical–chemical defense strategy of sea anemones, we have successfully developed a series of bioinspired fluorosilicone polyurethane coatings that integrate low-surface-energy physical antifouling and cationic antibacterial chemical antifouling. Among the four formulations, P-4 was identified as the optimal composition, exhibiting a fracture elongation of 78.19%, a normal adhesion strength of approximately 2.5 MPa, a water contact angle of 120°, and a surface free energy as low as 12.86 mN/m. Notably, the coating demonstrated excellent antibacterial activity against both Escherichia coli and Staphylococcus aureus, with inhibition rates exceeding 95%. Furthermore, the coating maintained stable hydrophobicity and low surface energy even after 72 h of water immersion, and its thermal decomposition occurred predominantly within 250–600 °C, indicating adequate thermal stability for typical marine service conditions. Mechanical evaluation revealed that P-4 possesses superior ductility (elongation at break 78.19%) while retaining sufficient hardness (2H) and adhesion strength. Collectively, the unique synergy of low surface energy, potent antibacterial action, robust mechanical properties, and thermal stability enables the P-4 coating to deliver long-term, reliable antifouling performance under static seawater conditions. This work not only validates the sea anemone-inspired dual-mechanism strategy but also provides a practical technological foundation for the engineering application and industrialization of green, durable marine antifouling coatings.

## Figures and Tables

**Table 1:** Hardness of a series of sea anemone-inspired fluorosilicone antifouling coatings.

Samples	P-1	P-2	P-3	P-4
Hardness	2H	2H	3H	2H

**Table 2:** Formulation design of the sea anemone-inspired fluorosilicone polyurethane coating resin.

Samples	PDMS-PU	PDMS-N+	TEOS	F-Resin	Dibutyltin Diacetate
P-1	80%	15%	5%	0%	0.08%
P-2	75%	15%	5%	5%	0.08%
P-3	65%	15%	5%	15%	0.08%
P-4	60%	30%	5%	5%	0.08%

## Data Availability

The data that support the findings of this study are available from the corresponding author upon reasonable request.
